# Odor‐Specific Symptoms in Parkinson’s Disease—UPSIT‐TC Odor Identification Status Versus Clinical Severity

**DOI:** 10.1155/padi/9572752

**Published:** 2026-10-22

**Authors:** Ting-Chun Lin, Lan-Yan Yang, Yu-Hsuan Lin, Hsin-Bei Lei, Shih-Chi Chiu, Ting-Chun Fang, Ming-Hong Chang, Yi-Jen Guo

**Affiliations:** ^1^ The Department of Neurological Institute Taichung Veterans General Hospital Taichung Taiwan vghtc.gov.tw; ^2^ Division of Clinical Trial Department of Medical Research Taichung Veterans General Hospital Taichung Taiwan vghtc.gov.tw; ^3^ Department of Post-Baccalaureate Medicine College of Medicine National Chung Hsing University Taichung Taiwan nchu.edu.tw; ^4^ Brain and Neuroscience Research Center College of Medicine National Chung Hsing University Taichung Taiwan nchu.edu.tw

**Keywords:** hyposmia, Parkinson’s disease, symptom-specific odors, UPSIT

## Abstract

Olfactory dysfunction is a well‐recognized prodromal sign of Parkinson’s disease (PD). This study aimed to explore the potential of short‐form olfactory tests as a more convenient method for assessing PD symptoms in clinical practice. This retrospective cross‐sectional study included 176 patients with PD. For each clinical measure, 40 separate logistic regression models evaluated its association with correct identification of each UPSIT‐TC odor. Odors showing nominal associations at unrounded *p* < 0.05 were designated candidate symptom‐associated odors. A symptom‐specific odor score was constructed when at least two odors (≥ 2) met this criterion. Associations of each clinical measure with the UPSIT‐TC total score and its corresponding symptom‐specific odor score were subsequently evaluated using correlation analyses and covariate‐adjusted regression models. Across all clinical measures, 101 nominal odor–clinical associations involving 31 unique odors were identified, with 17 clinical measures meeting the ≥ 2‐odor criterion. Symptom‐specific odor scores generally showed stronger unadjusted correlations with their corresponding clinical measures than did the UPSIT‐TC total score. After adjustment for age, sex, disease duration, and levodopa equivalent daily dose (LEDD), with false discovery rate correction across 32 models, symptom‐specific odor scores remained significantly associated with MDS‐UPDRS total and Parts I–III scores, bradykinesia, rigidity, axial symptoms, NMSS total and gastrointestinal and urinary domain scores, MoCA scores, and NPI total scores. In contrast, the UPSIT‐TC total score remained significantly associated only with rigidity. Symptom‐specific odor scores have the potential to serve as a more clinically practical tool for assessing PD symptom burden than the UPSIT‐TC total score. Further large‐scale, multicenter studies are needed to explore odors with higher specificity for PD clinical symptoms.

## 1. Introduction

Parkinson’s disease (PD) is a progressive neurodegenerative disorder that primarily affects motor control, although it also presents with a range of nonmotor symptoms. Olfactory dysfunction is a well‐recognized prodromal sign of PD, occurring in over 96% of patients [1], and it has been shown to have significant diagnostic importance in both clinical and research settings [2, 3]. Furthermore, the association between olfactory dysfunction and PD has been well established [4], and similar olfactory impairment has also been reported in several other neurodegenerative disorders, including Alzheimer’s disease and dementia with Lewy bodies [5]. These findings suggest that olfactory dysfunction represents a common manifestation of neurodegenerative processes.

Various methods have been developed to evaluate olfactory function, including the 40‐item University of Pennsylvania Smell Identification Test (UPSIT), the Sniffin’ Sticks test [6], and the Hyposmia Rating Scale [7]. Among these, the UPSIT is one of the most extensively used and validated measures for assessing olfactory identification performance [8, 9]. Many studies have used the UPSIT to investigate olfactory dysfunction, including its role in differentiating patients with PD from controls [10], and recent research has also explored the relationship between olfactory dysfunction and clinical symptoms such as motor deficits, cognitive impairment, and freezing of gait [11, 12].

Although several studies have reported the predictive value of the UPSIT for clinical symptoms [13], the full 40‐item test can be time‐consuming and exhausting for patients, especially when combined with multiple PD questionnaires in a single clinical visit. There is therefore a clinical need for a more streamlined olfactory examination with fewer test items that does not compromise the specificity of predicting clinical severity. A few studies have suggested that certain odors could serve as predictive markers for PD symptoms [11, 12, 14], while other studies have reported inconsistent results [15, 16].

This study aimed to investigate the associations between individual odor items on the Traditional Chinese version of UPSIT (UPSIT‐TC) and clinical symptoms of PD and to explore whether a short‐form olfactory test could offer a more practical approach for evaluating these associations in clinical practice.

## 2. Method

### 2.1. Participants

This retrospective cross‐sectional study included patients with PD who underwent olfactory testing and completed comprehensive PD questionnaires during routine annual clinical evaluations from October 2016 through April 2022. All participants fulfilled the International Parkinson and Movement Disorder Society criteria for clinically probable PD and were evaluated at the Center for Parkinson and Movement Disorders of Taichung Veterans General Hospital (VGHTC).

After completing the annual comprehensive motor and nonmotor symptom questionnaires, patients without prominent fatigue who were willing to undergo further evaluation received the UPSIT‐TC assessment. For patients who underwent multiple annual assessments, only data from the first evaluation were included in the analysis. Patients were excluded if their initial diagnosis was revised during subsequent follow‐up or if detailed item‐level data were incomplete during data review (Figure 1). The study cohort overlaps with that of our previous publication, in which UPSIT‐TC odors were grouped according to odor valence to examine their associations with clinical severity [17]. In contrast, the present analysis evaluates individual odor identification in relation to specific motor and nonmotor clinical domains.

**FIGURE 1:**
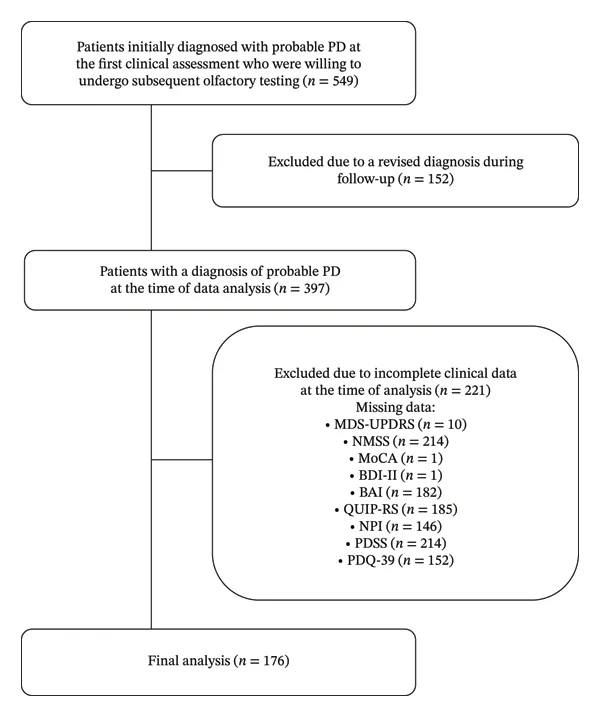
Flowchart of participant selection.

Demographic information including age, gender, disease duration, and levodopa equivalent daily dosage (LEDD) [18] was obtained from the patients and their families. As this retrospective study used de‐identified data from routine clinical evaluations and posed no more than minimal risk to participants, a waiver of informed consent was obtained and approved by the Institutional Review Board of VGHTC (Approval No. CE23443C). The study was conducted and reported in accordance with the STROBE guidelines.

### 2.2. Clinical Assessments

The patients were assessed while taking their regular medication. Motor manifestations were characterized using the Movement Disorder Society Unified Parkinson’s Disease Rating Scale (MDS‐UPDRS) and Hoehn and Yahr (H&Y) stage [19]. Four motor subscores were derived from MDS‐UPDRS Part III: bradykinesia (Items 3.2, 3.4–3.9, and 3.14), tremor (Items 3.15–3.18), rigidity (Item 3.3), and axial symptoms (Items 3.1 and 3.9–3.13) [20, 21]. Nonmotor symptom burden was quantified with the Non‐Motor Symptoms Scale (NMSS) [22], while cognitive performance was evaluated with the Montreal Cognitive Assessment (MoCA) [23], with permission obtained from the official copyright holder. Depression, anxiety, psychiatric disturbances, sleep quality, and impulsive–compulsive behaviors were assessed using the Beck Depression Inventory‐II (BDI‐II) [24], Beck Anxiety Inventory (BAI) [25], Neuropsychiatric Inventory (NPI) [26], Parkinson’s Disease Sleep Scale (PDSS) [27], and Questionnaire for Impulsive‐Compulsive Disorders in Parkinson’s Disease–Rating Scale (QUIP‐RS) [28], respectively. Quality of life was evaluated using the Parkinson’s Disease Questionnaire summary index (PDQ‐39 SI) [29].

All assessments were completed within approximately 2 hours. To minimize the potential effects of fatigue, participants were evaluated in the ON‐medication state. If a participant experienced substantial fatigue or was unable to complete all assessments during a single visit, the remaining evaluations were completed at a subsequent outpatient visit within 3 months.

### 2.3. Olfactory Testing

The UPSIT‐TC was adapted from the original UPSIT and validated by Jiang et al. [9]. It consists of 40 odorants presented on “scratch‐and‐sniff” labels. Participants used the tip of a pencil to release the odor from each label and then selected the correct response from four choices. To enhance cultural relevance for Taiwanese subjects, eight odorants in the original UPSIT were modified in the UPSIT‐TC. Specifically, cinnamon, cheddar cheese, gingerbread, dill pickle, grass, clove, wintergreen, and lime were replaced with sandalwood, fish, coffee, rubber tire, jasmine, grapefruit, magnolia, and lemon, respectively. Based on established UPSIT‐TC normative data, normosmia was defined as a UPSIT‐TC score of ≥ 30 in men aged 20–59 years, ≥ 24 in men aged 60–79 years, and ≥ 31 in women aged 20–59 years. Scores below these thresholds were classified as hyposmia [30]. The UPSIT‐TC has been used in a study to detect olfactory deficits in Taiwanese patients with early‐stage PD [13].

### 2.4. Statistical Analysis

All statistical analyses were performed using SPSS Version 22 (IBM Corporation, Armonk, New York, United States), and additional reproducible analyses were conducted using Python with the SciPy, statsmodels, and statsmodels.stats.multitest packages. Continuous variables are presented as mean ± standard deviation, and categorical variables are presented as number and percentage.

For each clinical measure, 40 separate logistic regression models were fitted with correct identification of each UPSIT‐TC odor (1 = *correct*, 0 = *incorrect*) as the dependent variable and the clinical score as the independent variable. Odors with nominal two‐sided unrounded *p* < 0.05 were designated candidate symptom‐associated odors. For each clinical measure, Benjamini–Hochberg false discovery rate (FDR) q values were additionally calculated across the 40 odor‐specific models to account for multiple testing. Age‐ and sex‐adjusted logistic regression models were performed as sensitivity analyses.

A symptom‐specific odor score was constructed for each clinical measure with at least two nominally associated odors (≥ 2). Correct identification of each qualifying odor contributed one point, whereas incorrect identification contributed zero points, with points summed across all qualifying odors. Pearson correlation coefficients were used to examine associations of each clinical measure with the UPSIT‐TC total score and its corresponding symptom‐specific odor score, except for H&Y stage, for which Spearman rank correlation was used. Partial correlation analyses were adjusted first for age and sex and subsequently for age, sex, disease duration, and LEDD. When LEDD itself was the clinical measure, the fully adjusted analysis included age, sex, and disease duration only to avoid adjustment for the variable itself. All primary unadjusted correlations involving the UPSIT‐TC total and symptom‐specific odor scores were treated as a single family of tests and corrected for multiple comparisons using the Benjamini–Hochberg FDR procedure.

Adjusted regression analyses were performed for each eligible clinical outcome. For each outcome, Model 1 included the UPSIT‐TC total score and Model 2 included the corresponding symptom‐specific odor score, with age, sex, disease duration, and LEDD included as covariates. Continuous outcomes were analyzed using multivariable linear regression, whereas H&Y stage was analyzed using ordinal logistic regression. LEDD was not analyzed as a regression outcome because it was included as a prespecified covariate. All models evaluating the UPSIT‐TC total and symptom‐specific odor scores were treated as a single family of tests and corrected for multiple comparisons using the Benjamini–Hochberg FDR procedure. Because symptom‐specific odor scores were derived and evaluated in the same study sample, these downstream association analyses were considered exploratory.

## 3. Results

A total of 549 patients who met the diagnostic criteria for probable PD and agreed to undergo olfactory testing were initially enrolled. During follow‐up, 152 patients were excluded because their initial diagnoses were revised. Among the remaining 397 patients, only data from the first assessment were retained for analysis. An additional 221 patients were excluded because of incomplete clinical data. Consequently, 176 patients were included in the final analysis (Figure 1). The average age of the participants was 67.1 ± 9.71 years, with 110 males and 66 females. The average disease duration was 4.69 ± 5.60 years. The mean score on the MDS‐UPDRS scale was 52.85 ± 26.32, and the average LEDD was 518.73 ± 445.14 mg/day. Detailed participant data are presented in Table 1.

**TABLE 1:** Demographic data of the PD patients.

	PD participants (*N* = 176)
Age, years (Mean ± SD)	67.10 ± 9.71
Male, *N* (%)	110 (62.5%)
Disease duration, years (Mean ± SD)	4.69 ± 5.60
LEDD, mg/day (Mean ± SD)	518.73 ± 445.14
MDS‐UPDRS score (Mean ± SD)	
Total	52.85 ± 26.32
Part I	10.51 ± 6.52
Part II	11.39 ± 9.25
Part III	29.55 ± 14.08
Bradykinesia	15.44 ± 8.05
Tremor	3.55 ± 3.69
Rigidity	7.11 ± 3.92
Axial	3.88 ± 3.88
Part IV	1.40 ± 3.20
H&Y stage (Mean ± SD)	2.18 ± 0.76
NMSS (Mean ± SD)	
Total	39.86 ± 31.43
Cardiovascular including falls	2.23 ± 4.01
Sleep/fatigue	8.65 ± 8.14
Mood/cognition	6.81 ± 9.13
Perceptual problems/hallucinations	0.77 ± 2.83
Attention/memory	3.76 ± 4.97
Gastrointestinal tract	5.41 ± 6.24
Urinary	7.01 ± 6.31
Sexual function	1.43 ± 3.17
Miscellaneous	3.80 ± 5.29
MoCA (Mean ± SD)	24.13 ± 5.24
BDI‐II (Mean ± SD)	12.11 ± 9.58
BAI (Mean ± SD)	10.19 ± 9.42
QUIP‐RS (Mean ± SD)	1.24 ± 4.53
NPI Total (Mean ± SD)	6.56 ± 7.74
PDSS (Mean ± SD)	110.60 ± 23.33
UPSIT‐TC (Mean ± SD)	16.06 ± 6.81
PDQ‐39 SI (Mean ± SD)	22.40 ± 18.49

*Note: N*, number of patients. H&Y stage, Hoehn and Yahr stage. MoCA, Montreal Cognitive Assessment. QUIP‐RS, Questionnaire for Impulsive‐Compulsive Disorders in Parkinson’s Disease–Rating Scale. UPSIT‐TC, Traditional Chinese version of the University of Pennsylvania Smell Identification Test. PDQ‐39 SI, 39‐item Parkinson’s Disease Questionnaire summary index. Bradykinesia was derived from MDS‐UPDRS Part III Items 3.2, 3.4–3.9, and 3.14; tremor from Items 3.15–3.18; rigidity from Item 3.3; and axial symptoms from Items 3.1 and 3.9–3.13.

Abbreviations: BAI, Beck Anxiety Inventory; BDI‐II, Beck Depression Inventory‐II; LEDD, levodopa equivalent daily dose; MDS‐UPDRS, Movement Disorder Society Unified Parkinson’s Disease Rating Scale; NMSS, Non‐Motor Symptoms Scale; NPI, Neuropsychiatric Inventory; PD, Parkinson’s disease; PDSS, Parkinson’s Disease Sleep Scale; SD, standard deviation.

### 3.1. Odors Associated With Motor and Nonmotor Severity

In exploratory logistic regression analyses, multiple individual UPSIT‐TC odor items showed nominal associations with clinical symptom severity across motor and nonmotor domains. Seven odor–symptom associations remained significant after FDR correction. Rubber tire remained significantly associated with MDS‐UPDRS total (OR: 0.976, *q* = 0.015), MDS‐UPDRS Part II (OR: 0.933, *q* = 0.023), rigidity (OR: 0.874, *q* = 0.022), and MoCA (OR: 1.128, *q* = 0.013). In addition, sandalwood was associated with MDS‐UPDRS Part II (OR: 0.944, *q* = 0.028), whereas jasmine (OR: 0.878, *q* = 0.037) and watermelon (OR: 0.866, *q* = 0.022) were associated with rigidity.

Several odors also recurred across multiple clinical measures. Among the 17 clinical measures eligible for construction of symptom‐specific odor scores, rubber tire was the most frequently included odor, appearing in 15 sets, followed by sandalwood in 10 sets, leather and peach in 9 sets each, and mint and smoke in 7 sets each. Specifically, rubber tire was included in the symptom‐specific odor sets for MDS‐UPDRS total and Parts I–III, rigidity and axial subscores, H&Y stage, NMSS total, gastrointestinal, urinary, and sexual function domains, MoCA, NPI total, PDSS, and PDQ‐39 SI.

For overall PD severity, higher MDS‐UPDRS total scores were associated with lower odds of correctly identifying pizza, bubble gum, mint, sandalwood, leather, cedar, rubber tire, peach, orange, and smoke. Similar inverse associations were observed for MDS‐UPDRS Part I with sandalwood, leather, rubber tire, and smoke, and for Part II with bubble gum, mint, sandalwood, leather, rubber tire, lilac, peach, orange, and smoke. Higher MDS‐UPDRS Part III scores were also related to lower odds of identifying pizza, bubble gum, mint, sandalwood, leather, rubber tire, peach, jasmine, watermelon, baby powder, and smoke. For the motor subscores, bradykinesia was related to bubble gum and mint; rigidity to pizza, menthol, motor oil, banana, sandalwood, leather, onion, rubber tire, turpentine, peach, jasmine, watermelon, paint thinner, smoke, natural gas, and peanut; and axial symptoms to menthol, mint, sandalwood, licorice, rubber tire, and orange. Higher H&Y stage was associated with lower odds of identifying sandalwood, leather, cedar, and rubber tire, whereas higher LEDD showed an inverse relationship with identification of coconut, lilac, and peach.

For nonmotor symptoms, higher NMSS total scores were associated with lower odds of identifying rubber tire, peach, smoke, and soap. Similar associations were observed for the gastrointestinal domain with mint, leather, coconut, cedar, rubber tire, and peach, and the urinary domain with fish, chocolate, rubber tire, and peach. In the sexual function domain, sandalwood, leather, and rubber tire showed inverse associations, whereas lemon showed a positive association. Sleep/fatigue was related to rubber tire, whereas the perceptual domain was related to coffee. Higher MoCA scores were associated with greater odds of correctly identifying bubble gum, menthol, mint, sandalwood, rubber tire, peach, pineapple, and smoke. Among the remaining measures, NPI total was related to licorice and rubber tire, QUIP‐RS to pine, PDSS to leather and rubber tire, and PDQ‐39 SI to sandalwood, cedar, and rubber tire. The corresponding ORs, nominal p values, FDR‐adjusted q values, and age‐ and sex‐adjusted p values are presented in Supporting Table 1.

### 3.2. Comparisons of Correlations Between UPSIT‐TC Total Score and Symptom‐Specific Odor Scores With PD Clinical Symptoms

Odors showing nominal associations with individual PD clinical measures were defined as symptom‐specific odors. For each clinical measure associated with at least two odor items, a symptom‐specific odor score was constructed by assigning one point for each correctly identified symptom‐specific odor. Of the 31 unique odors showing nominal associations with PD clinical measures, 29 were included in at least one symptom‐specific odor score (Tables 2 and 3).

**TABLE 2:** Key associations between clinical symptom severity and individual UPSIT‐TC odor identification.

Clinical domain	Symptom severity score	Associated odor item	OR for correct odor identification	*p* value	*q* value	Age/sex‐adjusted *p* value
Overall motor/nonmotor severity	MDS‐UPDRS Total	Pizza	0.981	0.032	0.162	0.024
Overall motor/nonmotor severity	MDS‐UPDRS Total	Bubble gum	0.986	0.037	0.162	0.079
Overall motor/nonmotor severity	MDS‐UPDRS Total	Mint	0.986	0.020	0.134	0.042
Overall motor/nonmotor severity	MDS‐UPDRS Total	Sandalwood	0.982	0.005	0.070	0.040
Overall motor/nonmotor severity	MDS‐UPDRS Total	Leather	0.983	0.008	0.070	0.037
Overall motor/nonmotor severity	MDS‐UPDRS Total	Cedar	0.986	0.041	0.162	0.077
Overall motor/nonmotor severity	MDS‐UPDRS Total	Rubber tire	0.976	< 0.001	0.015	0.004
Overall motor/nonmotor severity	MDS‐UPDRS Total	Peach	0.981	0.007	0.070	0.024
Overall motor/nonmotor severity	MDS‐UPDRS Total	Orange	0.985	0.036	0.162	0.050
Overall motor/nonmotor severity	MDS‐UPDRS Total	Smoke	0.982	0.009	0.070	0.032
Nonmotor experiences of daily living	MDS‐UPDRS Part I	Sandalwood	0.951	0.042	0.418	0.128
Nonmotor experiences of daily living	MDS‐UPDRS Part I	Leather	0.945	0.024	0.316	0.067
Nonmotor experiences of daily living	MDS‐UPDRS Part I	Rubber tire	0.926	0.003	0.110	0.007
Nonmotor experiences of daily living	MDS‐UPDRS Part I	Smoke	0.943	0.023	0.316	0.050
Motor experiences of daily living	MDS‐UPDRS Part II	Bubble gum	0.948	0.010	0.081	0.024
Motor experiences of daily living	MDS‐UPDRS Part II	Mint	0.965	0.043	0.189	0.088
Motor experiences of daily living	MDS‐UPDRS Part II	Sandalwood	0.944	0.001	0.028	0.015
Motor experiences of daily living	MDS‐UPDRS Part II	Leather	0.956	0.016	0.095	0.072
Motor experiences of daily living	MDS‐UPDRS Part II	Rubber tire	0.933	< 0.001	0.023	0.006
Motor experiences of daily living	MDS‐UPDRS Part II	Lilac	0.959	0.017	0.095	0.014
Motor experiences of daily living	MDS‐UPDRS Part II	Peach	0.944	0.006	0.068	0.023
Motor experiences of daily living	MDS‐UPDRS Part II	Orange	0.939	0.007	0.068	0.009
Motor experiences of daily living	MDS‐UPDRS Part II	Smoke	0.960	0.033	0.167	0.115
Motor examination	MDS‐UPDRS Part III	Pizza	0.966	0.028	0.149	0.028
Motor examination	MDS‐UPDRS Part III	Bubble gum	0.974	0.031	0.149	0.078
Motor examination	MDS‐UPDRS Part III	Mint	0.974	0.024	0.149	0.043
Motor examination	MDS‐UPDRS Part III	Sandalwood	0.977	0.041	0.149	0.236
Motor examination	MDS‐UPDRS Part III	Leather	0.977	0.041	0.149	0.140
Motor examination	MDS‐UPDRS Part III	Rubber tire	0.968	0.006	0.149	0.057
Motor examination	MDS‐UPDRS Part III	Peach	0.972	0.020	0.149	0.055
Motor examination	MDS‐UPDRS Part III	Jasmine	0.974	0.036	0.149	0.058
Motor examination	MDS‐UPDRS Part III	Watermelon	0.968	0.011	0.149	0.025
Motor examination	MDS‐UPDRS Part III	Baby powder	0.976	0.034	0.149	0.093
Motor examination	MDS‐UPDRS Part III	Smoke	0.973	0.022	0.149	0.074
Motor examination	Bradykinesia	Bubble gum	0.948	0.012	0.365	0.022
Motor examination	Bradykinesia	Mint	0.961	0.044	0.365	0.070
Motor examination	Rigidity	Pizza	0.884	0.015	0.086	0.019
Motor examination	Rigidity	Menthol	0.922	0.041	0.119	0.086
Motor examination	Rigidity	Motor oil	0.916	0.048	0.119	0.085
Motor examination	Rigidity	Banana	0.922	0.044	0.119	0.060
Motor examination	Rigidity	Sandalwood	0.917	0.039	0.119	0.150
Motor examination	Rigidity	Leather	0.916	0.027	0.119	0.061
Motor examination	Rigidity	Onion	0.923	0.047	0.119	0.131
Motor examination	Rigidity	Rubber tire	0.874	0.001	0.022	0.009
Motor examination	Rigidity	Turpentine	0.894	0.006	0.057	0.014
Motor examination	Rigidity	Peach	0.900	0.011	0.070	0.017
Motor examination	Rigidity	Jasmine	0.878	0.003	0.037	0.005
Motor examination	Rigidity	Watermelon	0.866	< 0.001	0.022	0.002
Motor examination	Rigidity	Paint thinner	0.921	0.042	0.119	0.054
Motor examination	Rigidity	Smoke	0.921	0.040	0.119	0.092
Motor examination	Rigidity	Natural gas	0.919	0.034	0.119	0.073
Motor examination	Rigidity	Peanut	0.896	0.009	0.070	0.009
Motor examination	Axial	Menthol	0.919	0.041	0.321	0.049
Motor examination	Axial	Mint	0.912	0.033	0.321	0.070
Motor examination	Axial	Sandalwood	0.914	0.028	0.321	0.209
Motor examination	Axial	Licorice	0.917	0.048	0.321	0.056
Motor examination	Axial	Rubber tire	0.902	0.017	0.321	0.127
Motor examination	Axial	Orange	0.880	0.017	0.321	0.019
Disease stage	H&Y stage	Sandalwood	0.585	0.013	0.265	0.163
Disease stage	H&Y stage	Leather	0.621	0.029	0.285	0.134
Disease stage	H&Y stage	Cedar	0.572	0.022	0.285	0.049
Disease stage	H&Y stage	Rubber tire	0.560	0.009	0.265	0.119
Medication burden	LEDD	Coconut	0.999	0.023	0.368	0.018
Medication burden	LEDD	Lilac	0.999	0.014	0.368	0.010
Medication burden	LEDD	Peach	0.999	0.028	0.368	0.016
Cognition	MoCA	Bubble gum	1.097	0.007	0.059	0.012
Cognition	MoCA	Menthol	1.075	0.017	0.096	0.006
Cognition	MoCA	Mint	1.104	0.003	0.050	0.007
Cognition	MoCA	Sandalwood	1.088	0.006	0.059	0.217
Cognition	MoCA	Rubber tire	1.128	< 0.001	0.013	0.010
Cognition	MoCA	Peach	1.101	0.006	0.059	0.049
Cognition	MoCA	Pineapple	1.077	0.032	0.162	0.320
Cognition	MoCA	Smoke	1.082	0.017	0.096	0.111
Nonmotor symptom burden	NMSS Total	Rubber tire	0.986	0.008	0.321	0.024
Nonmotor symptom burden	NMSS Total	Peach	0.987	0.030	0.455	0.074
Nonmotor symptom burden	NMSS Total	Smoke	0.989	0.042	0.455	0.092
Nonmotor symptom burden	NMSS Total	Soap	0.990	0.046	0.455	0.036
Nonmotor symptom domain	NMSS Gastrointestinal	Mint	0.938	0.019	0.193	0.025
Nonmotor symptom domain	NMSS Gastrointestinal	Leather	0.947	0.043	0.290	0.077
Nonmotor symptom domain	NMSS Gastrointestinal	Coconut	0.923	0.044	0.290	0.044
Nonmotor symptom domain	NMSS Gastrointestinal	Cedar	0.926	0.019	0.193	0.028
Nonmotor symptom domain	NMSS Gastrointestinal	Rubber tire	0.918	0.003	0.106	0.008
Nonmotor symptom domain	NMSS Gastrointestinal	Peach	0.928	0.015	0.193	0.024
Nonmotor symptom domain	NMSS Urinary	Fish	0.812	0.020	0.371	0.027
Nonmotor symptom domain	NMSS Urinary	Chocolate	0.950	0.041	0.371	0.065
Nonmotor symptom domain	NMSS Urinary	Rubber tire	0.945	0.028	0.371	0.105
Nonmotor symptom domain	NMSS Urinary	Peach	0.924	0.010	0.371	0.025
Nonmotor symptom domain	NMSS Sexual	Sandalwood	0.906	0.050	0.435	0.172
Nonmotor symptom domain	NMSS Sexual	Leather	0.891	0.048	0.435	0.094
Nonmotor symptom domain	NMSS Sexual	Rubber tire	0.836	0.006	0.254	0.034
Nonmotor symptom domain	NMSS Sexual	Lemon	1.135	0.015	0.297	0.012
Neuropsychiatric symptoms	NPI Total	Licorice	0.952	0.033	0.653	0.039
Neuropsychiatric symptoms	NPI Total	Rubber tire	0.941	0.007	0.281	0.018
Sleep quality	PDSS	Leather	1.022	0.003	0.115	0.009
Sleep quality	PDSS	Rubber tire	1.017	0.014	0.283	0.033
Quality of life	PDQ‐39 SI	Sandalwood	0.980	0.021	0.424	0.050
Quality of life	PDQ‐39 SI	Cedar	0.980	0.043	0.550	0.051
Quality of life	PDQ‐39 SI	Rubber tire	0.977	0.010	0.394	0.014

*Note:* Values are odds ratios estimated from logistic regression models examining the association between each clinical severity score and correct identification of each UPSIT‐TC odor item. OR < 1 indicates that a one‐unit increase in the clinical measure was associated with lower odds of correct odor identification, whereas OR > 1 indicates greater odds of correct odor identification. The q values were calculated using the Benjamini–Hochberg false discovery rate procedure across all 40 odor‐specific models for each clinical measure; full results are presented in Supporting Table 1. Age/sex‐adjusted p values were obtained from models additionally adjusted for age and sex. This analysis was exploratory and should be interpreted as cross‐sectional associations rather than longitudinal prediction. UPSIT‐TC, Traditional Chinese version of the University of Pennsylvania Smell Identification Test. H&Y stage, Hoehn and Yahr stage. PDQ‐39 SI, 39‐item Parkinson’s Disease Questionnaire summary index; Bradykinesia subscore, MDS‐UPDRS Part III Items 3.2, 3.4–3.9, and 3.14; Rigidity subscore, MDS‐UPDRS Part III Item 3.3; Axial subscore, MDS‐UPDRS Part III Items 3.1 and 3.9–3.13. MoCA, Montreal Cognitive Assessment.

Abbreviations: LEDD, levodopa equivalent daily dose; MDS‐UPDRS, Movement Disorder Society Unified Parkinson’s Disease Rating Scale; NMSS, Non‐Motor Symptoms Scale; NPI, Neuropsychiatric Inventory; OR, Odds ratio; PDSS, Parkinson’s Disease Sleep Scale.

**TABLE 3:** Selected odors used to construct each PD symptom‐specific odor score.

Clinical measure	Number of odors	Included odors
MDS‐UPDRS Total score	10	Pizza, bubble gum, mint, sandalwood, leather, cedar, rubber tire, peach, orange, smoke
MDS‐UPDRS Part I score	4	Sandalwood, leather, rubber tire, smoke
MDS‐UPDRS Part II score	9	Bubble gum, mint, sandalwood, leather, rubber tire, lilac, peach, orange, smoke
MDS‐UPDRS Part III score	11	Pizza, bubble gum, mint, sandalwood, leather, rubber tire, peach, jasmine, watermelon, baby powder, smoke
Bradykinesia subscore	2	Bubble gum, mint
Rigidity subscore	16	Pizza, menthol, motor oil, banana, sandalwood, leather, onion, rubber tire, turpentine, peach, jasmine, watermelon, paint thinner, smoke, natural gas, peanut
Axial subscore	6	Menthol, mint, sandalwood, licorice, rubber tire, orange
H&Y stage	4	Sandalwood, leather, cedar, rubber tire
LEDD	3	Coconut, lilac, peach
MoCA	8	Bubble gum, menthol, mint, sandalwood, rubber tire, peach, pineapple, smoke
NMSS Total	4	Rubber tire, peach, smoke, soap
NMSS GI	6	Mint, leather, coconut, cedar, rubber tire, peach
NMSS Urinary	4	Fish, chocolate, rubber tire, peach
NMSS Sexual	4	Sandalwood, leather, rubber tire, lemon
NPI Total	2	Licorice, rubber tire
PDSS	2	Leather, rubber tire
PDQ‐39 SI	3	Sandalwood, cedar, rubber tire

*Note:* Composition of the symptom‐specific odor scoring systems showing the odor items included for each PD clinical symptom. H&Y stage, Hoehn and Yahr stage. NMSS GI, NMSS Gastrointestinal tract; NMSS Sexual, Sexual function; MoCA, Montreal Cognitive Assessment. UPSIT‐TC, Traditional Chinese version of the University of Pennsylvania Smell Identification Test; PDQ‐39 SI, 39‐item Parkinson’s Disease Questionnaire summary index; Bradykinesia subscore, MDS‐UPDRS Part III Items 3.2, 3.4–3.9, and 3.14; Rigidity subscore, MDS‐UPDRS Part III Item 3.3; Axial subscore, MDS‐UPDRS Part III Items 3.1 and 3.9–3.13.

Abbreviations: LEDD, levodopa equivalent daily dose; MDS‐UPDRS, Movement Disorder Society Unified Parkinson’s Disease Rating Scale; NMSS, Non‐Motor Symptoms Scale; NPI, Neuropsychiatric Inventory; PD, Parkinson’s disease; PDSS, Parkinson’s Disease Sleep Scale.

Correlations of clinical measures with the UPSIT‐TC total score and the corresponding symptom‐specific odor scores were examined (Figure 2). Across the 17 eligible clinical measures, the symptom‐specific odor scores showed larger absolute correlation coefficients than the UPSIT‐TC total score. Significant correlations were observed for 16 of the 17 symptom‐specific odor scores after FDR correction, including MDS‐UPDRS total (*r* = −0.403, *p* < 0.001), Part I (*r* = −0.264, *p* < 0.001), Part II (*r* = −0.400, *p* < 0.001), Part III (*r* = −0.342, *p* < 0.001), bradykinesia (*r* = −0.227, *p* = 0.002), rigidity (*r* = −0.401, *p* < 0.001), axial symptoms (*r* = −0.307, *p* < 0.001), H&Y stage (*ρ* = −0.283, *p* < 0.001), LEDD (*r* = −0.263, *p* < 0.001), MoCA (*r* = 0.410, *p* < 0.001), NMSS total (*r* = −0.266, *p* < 0.001), NMSS gastrointestinal tract (*r* = −0.326, *p* < 0.001), NMSS urinary (*r* = −0.281, *p* < 0.001), NPI total (*r* = −0.252, *p* < 0.001), PDSS (*r* = 0.257, *p* < 0.001), and PDQ‐39 SI (*r* = −0.252, *p* < 0.001). The association with NMSS sexual function did not reach statistical significance (*r* = −0.139, *p* = 0.067). These associations generally remained significant in covariate‐adjusted analyses (Supporting Table 2).

**FIGURE 2:**
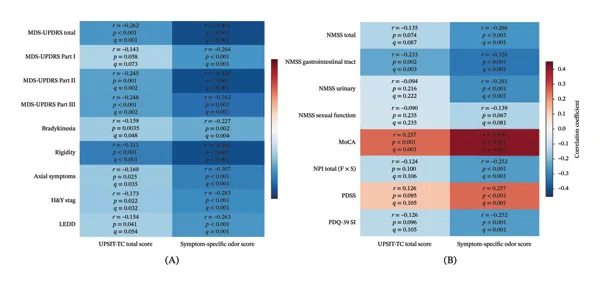
Heatmaps show the unadjusted associations of the UPSIT‐TC total score and corresponding symptom‐specific odor scores with clinical measures. (A) Motor and overall clinical severity measures, including MDS‐UPDRS total and Parts I–III, bradykinesia, rigidity, axial symptoms, H&Y stage, and LEDD. (B) Nonmotor, cognitive, neuropsychiatric, sleep, and quality‐of‐life measures, including NMSS total and gastrointestinal, urinary, and sexual domains, MoCA, NPI total, PDSS, and PDQ‐39 SI. Pearson correlation coefficients (*r*) were used for all measures except H&Y stage, for which Spearman rank correlation (ρ) was used. Each cell displays the correlation coefficient, two‐sided *p* value, and Benjamini–Hochberg false‐discovery‐rate–adjusted *q* value. FDR correction was applied across all primary unadjusted correlations involving the two olfactory scores. Bold text indicates associations remaining significant after FDR correction (*q* < 0.05). Color intensity represents the magnitude and direction of the correlation, with red indicating positive and blue indicating negative associations. UPSIT‐TC, Traditional Chinese version of the University of Pennsylvania Smell Identification Test; MDS‐UPDRS, Movement Disorder Society ‐Unified Parkinson’s Disease Rating Scale; Bradykinesia subscore, MDS‐UPDRS Part III items 3.2, 3.4–3.9, and 3.14; Rigidity subscore, MDS‐UPDRS Part III item 3.3; Axial subscore, MDS‐UPDRS Part III items 3.1 and 3.9–3.13; H&Y, Hoehn and Yahr; LEDD, levodopa equivalent daily dose; NMSS, Non‐Motor Symptoms Scale; MoCA, Montreal Cognitive Assessment; NPI, Neuropsychiatric Inventory; PDSS, Parkinson’s Disease Sleep Scale; PDQ‐39 SI, 39‐item Parkinson’s Disease Questionnaire Summary Index.

Multivariable linear regression analyses were then performed to examine the adjusted associations of the UPSIT‐TC total score (Model 1) and the corresponding symptom‐specific odor score (Model 2) with clinical severity (Table 4). Continuous outcomes were analyzed using linear regression, whereas H&Y stage was analyzed using ordinal logistic regression. Both models were adjusted for age, sex, disease duration, and LEDD, with the respective olfactory score included as the predictor of interest. After FDR correction, symptom‐specific odor scores remained independently associated with 12 of the 16 clinical outcomes examined: MDS‐UPDRS total (*B* = −3.100, 95% CI: −4.711 to −1.488, *q* = 0.002), Part I (*B* = −0.846, 95% CI: −1.538 to −0.154, *q* = 0.041), Part II (*B* = −0.990, 95% CI: −1.545 to −0.435, *q* = 0.004), Part III (*B* = −1.404, 95% CI: −2.198 to −0.610, *q* = 0.004), bradykinesia (*B* = −2.014, 95% CI: −3.586 to −0.443, *q* = 0.033), rigidity (*B* = −0.409, 95% CI: −0.572 to −0.246, *q* < 0.001), axial symptoms (*B* = −0.480, 95% CI: −0.822 to −0.137, *q* = 0.022), MoCA (*B* = 0.715, 95% CI: 0.377 to 1.052, *q* = 0.001), NMSS total (*B* = −4.635, 95% CI: −8.183 to −1.087, *q* = 0.032), NMSS gastrointestinal tract (*B* = −0.913, 95% CI: −1.479 to −0.347, *q* = 0.008), NMSS urinary (*B* = −1.305, 95% CI: −2.206 to −0.405, *q* = 0.019), and NPI total (*B* = −1.920, 95% CI: −3.395 to −0.445, *q* = 0.032). By contrast, the UPSIT‐TC total score remained significant after FDR correction only for rigidity (*B* = −0.152, 95% CI: −0.240 to −0.064, *q* = 0.004). Thus, symptom‐specific odor scores showed FDR‐significant adjusted associations with a broader range of clinical measures than the UPSIT‐TC total score.

**TABLE 4:** Adjusted associations of olfactory scores with clinical severity scores.

Clinical domain	Clinical severity score	Model	Olfactory score	*N*	Adjusted B (95% CI)	*p* value	*q* value	Adjusted *R* ^2^	∆ adjusted *R* ^2^	AIC
Overall severity	MDS‐UPDRS Total	Model 1	UPSIT‐TC total score	176	−0.531 (−1.078 to 0.015)	0.057	0.096	0.222	0.012	1612.368
Overall severity	MDS‐UPDRS Total	Model 2	Symptom‐specific odor score	176	−3.100 (−4.711 to −1.488)	< 0.001	0.002	0.268	0.057	1601.807
Nonmotor	MDS‐UPDRS Part I	Model 1	UPSIT‐TC total score	176	−0.047 (−0.187 to 0.093)	0.506	0.675	0.170	−0.003	1132.623
Nonmotor	MDS‐UPDRS Part I	Model 2	Symptom‐specific odor score	176	−0.846 (−1.538 to −0.154)	0.017	0.041	0.195	0.023	1127.149
Motor	MDS‐UPDRS Part II	Model 1	UPSIT‐TC total score	176	−0.141 (−0.326 to 0.043)	0.132	0.201	0.282	0.005	1230.198
Motor	MDS‐UPDRS Part II	Model 2	Symptom‐specific odor score	176	−0.990 (−1.545 to −0.435)	< 0.001	0.004	0.321	0.045	1220.151
Motor	MDS‐UPDRS Part III	Model 1	UPSIT‐TC total score	176	−0.330 (−0.646 to −0.013)	0.041	0.082	0.091	0.017	1419.642
Motor	MDS‐UPDRS Part III	Model 2	Symptom‐specific odor score	176	−1.404 (−2.198 to −0.610)	< 0.001	0.004	0.130	0.057	1411.794
Motor	Bradykinesia	Model 1	UPSIT‐TC total score	176	−0.105 (−0.291 to 0.080)	0.263	0.366	0.043	0.001	1231.715
Motor	Bradykinesia	Model 2	Symptom‐specific odor score	176	−2.014 (−3.586 to −0.443)	0.012	0.033	0.071	0.029	1226.51
Motor	Rigidity	Model 1	UPSIT‐TC total score	176	−0.152 (−0.240 to −0.064)	< 0.001	0.004	0.100	0.057	967.694
Motor	Rigidity	Model 2	Symptom‐specific odor score	176	−0.409 (−0.572 to −0.246)	< 0.001	< 0.001	0.159	0.116	955.714
Motor	Axial	Model 1	UPSIT‐TC total score	176	−0.022 (−0.106 to 0.061)	0.600	0.711	0.167	−0.004	950.498
Motor	Axial	Model 2	Symptom‐specific odor score	176	−0.480 (−0.822 to −0.137)	0.006	0.022	0.202	0.031	943.038
Disease stage	H&Y stage	Model 1	UPSIT‐TC total score	176	−0.014 (−0.062 to 0.035)	0.578	0.711	0.094	0.001	356.154
Disease stage	H&Y stage	Model 2	Symptom‐specific odor score	176	−0.249 (−0.517 to 0.019)	0.069	0.111	0.103	0.009	353.081
Cognition	MoCA	Model 1	UPSIT‐TC total score	176	0.111 (0.010–0.213)	0.032	0.068	0.325	0.015	1019.512
Cognition	MoCA	Model 2	Symptom‐specific odor score	176	0.715 (0.377–1.052)	< 0.001	0.001	0.371	0.061	1007.087
Nonmotor	NMSS Total	Model 1	UPSIT‐TC total score	176	−0.149 (−0.830 to 0.531)	0.665	0.760	0.155	−0.004	1689.386
Nonmotor	NMSS Total	Model 2	Symptom‐specific odor score	176	−4.635 (−8.183 to −1.087)	0.011	0.032	0.186	0.027	1682.827
Nonmotor	NMSS Gastrointestinal tract	Model 1	UPSIT‐TC total score	176	−0.137 (−0.274 to 0.000)	0.050	0.091	0.129	0.015	1125.494
Nonmotor	NMSS Gastrointestinal tract	Model 2	Symptom‐specific odor score	176	−0.913 (−1.479 to −0.347)	0.002	0.008	0.159	0.045	1119.278
Nonmotor	NMSS Urinary	Model 1	UPSIT‐TC total score	176	−0.017 (−0.163 to 0.128)	0.814	0.840	0.041	−0.005	1146.372
Nonmotor	NMSS Urinary	Model 2	Symptom‐specific odor score	176	−1.305 (−2.206 to −0.405)	0.005	0.019	0.085	0.038	1138.152
Nonmotor	NMSS Sexual function	Model 1	UPSIT‐TC total score	176	−0.011 (−0.085 to 0.063)	0.767	0.819	0.025	−0.005	907.229
Nonmotor	NMSS Sexual function	Model 2	Symptom‐specific odor score	176	−0.238 (−0.643 to 0.166)	0.246	0.358	0.032	0.002	905.923
Neuropsychiatric	NPI Total	Model 1	UPSIT‐TC total score	176	−0.050 (−0.219 to 0.120)	0.565	0.711	0.131	−0.003	1200.997
Neuropsychiatric	NPI Total	Model 2	Symptom‐specific odor score	176	−1.920 (−3.395 to −0.445)	0.011	0.032	0.162	0.027	1194.637
Sleep quality	PDSS	Model 1	UPSIT‐TC total score	176	0.091 (−0.409–0.590)	0.721	0.795	0.174	−0.004	1580.414
Sleep quality	PDSS	Model 2	Symptom‐specific odor score	176	4.657 (0.562–8.751)	0.026	0.060	0.197	0.019	1575.404
Quality of life	PDQ‐39 SI	Model 1	UPSIT‐TC total score	176	−0.037 (−0.402 to 0.328)	0.842	0.842	0.296	−0.004	1470.426
Quality of life	PDQ‐39 SI	Model 2	Symptom‐specific odor score	176	−2.432 (−4.878 to 0.014)	0.051	0.091	0.312	0.011	1466.522

*Note:* Values are regression coefficients from multivariable linear regression, except for H&Y stage, which was analyzed using ordinal logistic regression. All models were adjusted for age, sex, disease duration, and levodopa equivalent daily dose. Model 1 included the UPSIT‐TC total score as the olfactory score, whereas Model 2 included the corresponding symptom‐specific odor score. Adjusted B represents the mean change in the clinical severity score per one‐point increase in the olfactory score. *q* values were calculated using the Benjamini–Hochberg false discovery rate procedure across the primary olfactory score models shown in Table 4. ∆ adjusted *R* ^2^ represents the incremental explained variance after adding the olfactory score to a covariate‐only model. A lower AIC indicates better relative model fit. These analyses should be interpreted as cross‐sectional adjusted associations rather than longitudinal prediction. Bradykinesia subscore, MDS‐UPDRS Part III Items 3.2, 3.4–3.9, and 3.14; Rigidity subscore, MDS‐UPDRS Part III Item 3.3; Axial subscore, MDS‐UPDRS Part III Items 3.1 and 3.9–3.13; H&Y stage, Hoehn and Yahr stage; MoCA, Montreal Cognitive Assessment. PDQ‐39 SI, 39‐item Parkinson’s Disease Questionnaire summary index; UPSIT‐TC, Traditional Chinese version of the University of Pennsylvania Smell Identification Test.

Abbreviations: MDS‐UPDRS, Movement Disorder Society Unified Parkinson’s Disease Rating Scale; NMSS, Non‐Motor Symptoms Scale; NPI, Neuropsychiatric Inventory; PDSS, Parkinson’s Disease Sleep Scale.

## 4. Discussion

The present study suggests that the clinical relevance of olfactory dysfunction in PD may extend beyond the total UPSIT‐TC score. Although global olfactory performance was associated with several motor and nonmotor manifestations, item‐level analyses revealed that selected odors were preferentially related to specific clinical domains. This odor‐specific pattern suggests that deficits in identifying certain odors may provide information about clinically relevant dimensions of PD severity beyond that reflected by global olfactory performance. Moreover, when odors associated with specific clinical manifestations were grouped into symptom‐specific odor scores, their associations with PD severity were comparable to, and in some domains more robust than, those observed with the total UPSIT‐TC score. These findings support the potential utility of selected UPSIT‐TC odors as a brief, symptom‐oriented olfactory measure for assessing clinical severity in PD.

This interpretation is consistent with the heterogeneous neurobiological basis of olfactory dysfunction in PD. Alterations in acetylcholine, dopamine, norepinephrine, and serotonin across different brain regions have been proposed to contribute to olfactory impairment in PD [31]. Previous studies have reported associations between olfactory dysfunction and clinical manifestations, such as motor impairment, cognitive decline, constipation, sleep disturbance, and overall nonmotor symptom burden [11, 12, 14]. However, other studies have failed to demonstrate significant correlations between olfactory function and clinical severity [15, 16], suggesting that total olfactory scores may not consistently capture the symptom‐specific heterogeneity of PD. In this context, our item‐level approach may help explain these inconsistent findings by showing that selected odors, rather than the overall UPSIT‐TC score, were associated with specific clinical measures. Notably, several odors were repeatedly identified across multiple clinical measures, suggesting that certain odor identification items may reflect broader dimensions of PD symptom burden rather than being restricted to a single clinical domain. Rubber tire was the most frequently identified candidate odor, appearing in the symptom‐specific odor sets of 15 of the 17 eligible clinical measures, followed by sandalwood (10 measures), leather and peach (9 measures each), and mint and smoke (7 measures each). The recurrent involvement of these odors across measures of motor and nonmotor symptoms, cognition, neuropsychiatric manifestations, sleep, and quality of life suggests that they may represent particularly informative candidate items for the future development of abbreviated or symptom‐oriented olfactory assessments.

While most previous investigations have focused on developing short versions of olfactory tests to differentiate PD patients from healthy individuals or those with atypical parkinsonism [32–34], developing shorter symptom‐specific odor tests to assess the severity of PD is also important. Sensitivity to food odor detection has been reported to be odor‐specific during the aging process, influenced by factors such as the complexity of chemical compositions, the trigeminal component of odors, and even central imaginative interpretation [35]. Since the trigeminal pathway is reported to be less impaired than the olfactory system in patients with PD, olfactory dysfunction is believed to be more directly associated with abnormalities in the central olfactory structures of the brain [31, 36]. A previous functional MRI study showed that pleasant and unpleasant olfactory stimuli evoke distinct neural responses, with lateral orbitofrontal cortex activation associated with unpleasant odors and medial orbitofrontal cortex activity linked to pleasant odors and facial attractiveness [37, 38]. These findings provide a possible explanation for the specificity observed in odor identification, suggesting that different odors may be processed in distinct brain regions, which could in turn be associated with the clinical severity of various PD symptoms observed in the present study.

Several previous studies have also suggested that selected odors may have clinical relevance in PD. Glover et al. proposed a 3‐odorant score (bubblegum, chocolate, and smoke) to differentiate between “freezers” and “nonfreezers” [12]. In addition, Fujio et al. found that PD patients who could only identify four or fewer of six odors (lumber, menthol, Japanese orange, household gas, Hinoki cypress, and condensed milk) were associated with a potential decline in Mini‐Mental State Examination scores within 3 years [39]. In our previous study, we also found that the identification of odors such as banana, peach, magnolia, and baby powder was linked to rapid motor progression [32]. In contrast to our previous study [17], which grouped UPSIT‐TC odors by valence to examine their association with clinical severity, the present study investigated individual odor identification status in relation to clinical symptoms. This approach may provide improved discriminatory ability for the development of a shortened olfactory test in the future. Moreover, cultural differences may influence the learning process and familiarity with odor identification, thereby contributing to variations in odor identification specificity and its association with different clinical symptoms of PD across ethnicities [40].

This study has several limitations. First, the absence of a control group limits our ability to determine whether the observed patterns of selective odor impairment are specific to PD. Nevertheless, the present findings suggest the potential clinical utility of a brief olfactory identification assessment for evaluating symptom severity among patients with an established diagnosis of PD. Second, all participants were of Chinese ethnicity, and cultural differences may influence odor familiarity and identification across populations [40, 41]. Furthermore, this study was conducted at a single tertiary referral center; therefore, the findings may not be fully generalizable to other ethnic and cultural populations or clinical settings. Further multicenter and multiethnic studies including appropriate control groups are warranted to validate these findings. Third, the average MoCA score (24.13 ± 5.24) in this study was relatively low. Although we could not exclude the possibility of including some patients with cognitive decline, the normative MoCA score for the Taiwanese population in the same age group was 23.80 ± 4.57 in a previous study [42], and cognitive function was included as an important investigational outcome in this study. Fourth, potential confounding factors, including education level, smoking status, sinonasal disease, and previous infections, were not accounted for in the current analyses. These factors may influence olfactory identification and should be systematically assessed and appropriately adjusted for in future prospective studies. Fifth, because this was a retrospective observational study, the underlying pathophysiological mechanisms could not be directly examined. Future studies incorporating functional MRI, olfactory‐evoked electrophysiological testing, neuropathological examination, or analyses of genetic polymorphisms may provide further insights into the relationship between selective odor identification deficits and clinical severity in PD. Recent studies have demonstrated the potential of integrating olfactory‐evoked electrophysiological signals with advanced analytical approaches for the objective assessment of neurodegenerative disorders [43, 44]. These emerging methods may complement conventional odor identification testing and facilitate a more comprehensive understanding of olfactory dysfunction in PD. Finally, the analyses were cross‐sectional, and the same dataset was used to derive and evaluate the symptom‐specific odor scores, which may introduce circularity and increase the risk of overfitting. Although these scores showed stronger correlations with several clinical outcomes than the complete UPSIT‐TC, the effect sizes were generally mild to moderate. Therefore, the findings should be interpreted as exploratory and should not be considered evidence of longitudinal prediction, causal relationships, or the clinical validity of the proposed short‐form scores. External validation, prospective replication, and appropriate cross‐validation will be required before these symptom‐specific odor combinations can be translated into practical short‐form olfactory assessments or clinical screening tools. Future studies with predefined clinically meaningful thresholds may further evaluate ROC/AUC, sensitivity, specificity, confidence intervals, and optimal cutoff values. In addition, recent advances in machine learning–based analysis of physiological responses to olfactory stimuli [44] demonstrate the potential of computational approaches for disease classification. Similar data‐driven methods may be explored in future PD studies to evaluate the performance of symptom‐specific odor combinations and support the development of clinically applicable short‐form olfactory assessments.

## 5. Conclusion

This is the first study to suggest the potential clinical value of a shortened olfactory identification assessment showing stronger associations with PD symptom severity than the complete UPSIT‐TC. Using a uniform ≥ 2‐odor eligibility rule, symptom‐specific odor scores were constructed for 17 clinically meaningful PD measures, and many of these scores showed stronger cross‐sectional associations with PD symptom severity than the complete UPSIT‐TC score. Such a brief assessment may reduce administration time and patient burden, thereby potentially facilitating a more convenient evaluation of motor and nonmotor symptom burden in clinical practice. However, the observed associations were generally mild to moderate, and the proposed symptom‐specific odor scores require further refinement and validation before clinical implementation. Future studies should evaluate these prespecified odor sets in independent, prospective cohorts and determine their reproducibility, incremental clinical value, and feasibility as abbreviated olfactory assessments. These efforts may support the development of a more time‐efficient olfactory assessment tool for evaluating clinical symptom burden in patients with PD.

## Author Contributions

Ting‐Chun Lin participated in study design, conducted the analysis, and drafted the manuscript. Lan‐Yan Yang conducted the data analysis and helped draft the manuscript. Yi‐Jen Guo conceptualized and designed the study and helped draft the manuscript. Ting‐Chun Fang was involved in study design, contributed to data analysis, and helped draft the manuscript. Ming‐Hong Chang assisted in data collection. Yu‐Hsuan Lin, Hsin‐Bei Lei, and Shih‐Chi Chiu participated in data collection and conducted the initial analysis.

## Funding

This study was funded by a grant from Taichung Veterans General Hospital (TCVGH‐1144501B).

## Disclosure

All authors read and approved the final version of the manuscript. Part of this work was previously presented as a conference abstract at the International Congress of Parkinson’s Disease and Movement Disorders 2024.

## Conflicts of Interest

The authors declare no conflicts of interest.

## Supporting Information

Additional supporting information can be found online in the Supporting Information section.

## Supporting information


**Supporting Information 1** Supporting Table 1. Full list of nominal associations between clinical symptom severity and individual UPSIT‐TC odor identification. Supporting Table 2. Correlations between clinical severity scores and olfactory scores (*N* = 176)

**Supporting Information 2** STROBE Statement—checklist of items that should be included in reports of observational studies.

## Data Availability

The data that support the findings of this study are available from the corresponding author upon reasonable request.
