# Supplementary material for: Odor‐Specific Symptoms in Parkinson’s Disease—UPSIT‐TC Odor Identification Status Versus Clinical Severity
Source: Parkinsons Dis. 2026 Oct 22;2026:9572752. doi: 10.1155/padi/9572752 (PMC13687933; doi:10.1155/padi/9572752)
Supplement: Supplementary file 1 [file PADI-2026-9572752-s001.docx]

STROBE Statement—checklist of items that should be included in reports of observational studies

|  | Item No. | Recommendation | Page  No. | Relevant text from manuscript |
| --- | --- | --- | --- | --- |
| **Title and abstract** | 1 | (*a*) Indicate the study’s design with a commonly used term in the title or the abstract | 1 | This retrospective cross-sectional study recruited PD patients from Taichung Veterans General Hospital between October 2016 and April 2022. |
|  |  | (*b*) Provide in the abstract an informative and balanced summary of what was done and what was found | 1 | This retrospective cross-sectional study recruited PD patients from Taichung Veterans General Hospital between October 2016 and April 2022. Logistic regression was used to assess the detection status of each University of Pennsylvania Smell Identification Test (UPSIT) odor and the severity of PD symptoms. Odors significantly associated with specific clinical symptoms were combined to calculate a symptom-specific odor score.  A total of 176 PD patients were enrolled. Seventeen odors showed significant correlations with the severity of PD symptoms. In Pearson’s correlation analysis, symptom-specific odor scores were more strongly correlated with clinical outcomes than UPSIT total score, including Movement Disorder Society Unified Parkinson's Disease Rating Scale (MDS-UPDRS) total (Pearson's r=-0.384, p value<0.001), part II (Pearson's r=-0.418, p value<0.001), and part III scores (Pearson's r=-0.286, p value<0.001), Montreal Cognitive Assessment (Pearson's r=0.396, p value<0.001), and Non-Motor Symptoms Scale (NMSS) gastrointestinal symptoms (Pearson's r=-0.299, p value<0.001). Linear regression analysis confirmed the significance of these findings. While MDS-UPDRS total score (B = -3.890, p < 0.001), MDS-UPDRS part II score (B = -1.836, p < 0.001), and NMSS gastrointestinal tract subscore (B = -1.347, p = 0.005) were shown to have significant predictive value in the symptom-specific odor score model, this was not observed in the UPSIT total score model. |
| Introduction | | | |  |
| Background/rationale | 2 | Explain the scientific background and rationale for the investigation being reported | 2 | Olfactory dysfunction is a well-recognized prodromal sign of PD, occurring in over 96% of patients [1], and it has been shown to have significant diagnostic importance in both clinical and research settings [2, 3].  Many studies have used the UPSIT to investigate olfactory dysfunction, including its role in differentiating patients with PD from controls [6], and recent research has also explored the relationship between olfactory dysfunction and clinical symptoms such as motor deficits, cognitive impairment, and freezing of gait [7, 8].  Although several studies have reported the predictive value of the UPSIT for clinical symptoms [9], the full 40-item test can be time-consuming and exhausting for patients, especially when combined with multiple PD questionnaires in a single clinical visit. There is therefore a clinical need for a more streamlined olfactory examination with fewer test items that does not compromise the specificity of predicting clinical severity. A few studies have suggested that certain odors could serve as predictive markers for PD symptoms [7, 8, 10], while other studies have reported inconsistent results [11, 12]. |
| Objectives | 3 | State specific objectives, including any prespecified hypotheses | 2 | This study aimed to evaluate the correlation between each UPSIT item and clinical PD symptoms to explore the potential of short-form olfactory tests as a more convenient way to predict PD symptoms in clinical practice. |
| Methods | | | |  |
| Study design | 4 | Present key elements of study design early in the paper | 2 | This retrospective cross-sectional study was conducted between October 2016 and April 2022. |
| Setting | 5 | Describe the setting, locations, and relevant dates, including periods of recruitment, exposure, follow-up, and data collection | 2 | This retrospective cross-sectional study included patients with PD who underwent olfactory testing and completed comprehensive PD questionnaires during routine annual clinical evaluations between October 2016 and April 2022. Patients who met the diagnostic criteria for clinically probable PD according to the International Parkinson and Movement Disorder Society Clinical Diagnostic Criteria for Parkinson’s Disease were recruited from the Center for Parkinson and Movement Disorders at Taichung Veterans General Hospital (VGHTC). |
| Participants | 6 | (*a*) *Cohort study*—Give the eligibility criteria, and the sources and methods of selection of participants. Describe methods of follow-up  *Case-control study*—Give the eligibility criteria, and the sources and methods of case ascertainment and control selection. Give the rationale for the choice of cases and controls  *Cross-sectional study*—Give the eligibility criteria, and the sources and methods of selection of participants | 2 | This retrospective cross-sectional study included patients with PD who underwent olfactory testing and completed comprehensive PD questionnaires during routine annual clinical evaluations between October 2016 and April 2022. Patients who met the diagnostic criteria for clinically probable PD according to the International Parkinson and Movement Disorder Society Clinical Diagnostic Criteria for Parkinson’s Disease were recruited from the Center for Parkinson and Movement Disorders at Taichung Veterans General Hospital (VGHTC).  After completing the annual comprehensive motor and non-motor symptom questionnaires, patients without prominent fatigue who were willing to undergo further evaluation received the UPSIT-TC assessment. For patients who underwent multiple annual assessments, only data from the first evaluation were included in the analysis. Patients were excluded if their initial diagnosis was revised during subsequent follow-up or if detailed item-level data were incomplete during data review (Table 1) |
|  |  | (*b*) *Cohort study*—For matched studies, give matching criteria and number of exposed and unexposed  *Case-control study*—For matched studies, give matching criteria and the number of controls per case |  | Not applicable |
| Variables | 7 | Clearly define all outcomes, exposures, predictors, potential confounders, and effect modifiers. Give diagnostic criteria, if applicable | 3 | Disease severity was evaluated using the Movement Disorder Society Unified Parkinson's Disease Rating Scale (MDS-UPDRS) and the Hoehn and Yahr (H&Y) Scale for motor severity [14]. The Non-Motor Symptoms Scale (NMSS) [15] was used to assess the severity of non-motor symptoms. The Montreal Cognitive Assessment (MoCA) [16] was used to assess cognitive function, with permission obtained from the official copyright holder. Beck Depression Inventory-II (BDI-II) [17] for depression, Beck Anxiety Inventory (BAI) [18] for anxiety, Neuropsychiatric Inventory (NPI) [19] for psychiatric disturbances, Parkinson’s Disease Sleep Scale (PDSS) [20] for sleep quality, and Questionnaire for Impulsive-Compulsive Disorders in Parkinson’s Disease–Rating Scale (QUIP-RS) [21] for impulsive-compulsive behaviors. The quality of life of the patients was assessed using the Parkinson’s Disease Questionnaire summary index (PDQ-39 SI) [22].  The MDS-UPDRS part III scores were categorized into the following subdomains: tremor (sum of MDS-UPDRS items 3.15–3.18), rigidity (MDS-UPDRS item 3.3), bradykinesia (sum of MDS-UPDRS items 3.2, 3.4–3.9, and 3.14), and axial symptoms (sum of MDS-UPDRS items 3.1 and 3.9–3.13).  When validating the UPSIT-TC, eight odors in the original UPSIT were replaced due to constraints in availability and familiarity of the odors in Taiwanese subjects. |
| Data sources/ measurement | 8* | For each variable of interest, give sources of data and details of methods of assessment (measurement). Describe comparability of assessment methods if there is more than one group | 2 | This retrospective cross-sectional study included patients with PD who underwent olfactory testing and completed comprehensive PD questionnaires during routine annual clinical evaluations between October 2016 and April 2022. |
| Bias | 9 | Describe any efforts to address potential sources of bias | 7 | First, selection bias may be present due to the single-center design and the inclusion of only patients with a confirmed diagnosis of PD without a control group for comparison. |
| Study size | 10 | Explain how the study size was arrived at | 4 | A total of 176 consecutive patients who met the inclusion criteria during the study period were included in the final analysis. |

Continued on next page

| Quantitative variables | 11 | Explain how quantitative variables were handled in the analyses. If applicable, describe which groupings were chosen and why | 3 | Odors showing a statistically significant odds ratio between their identification status and clinical scores were grouped as symptom-specific odors. The identification of a symptom-specific odor was scored as one, while misidentification of this smell was scored as zero. The symptom-specific odor score was calculated as the sum of the identification status of odors that showed a statistically significant odds ratio with the specific clinical score. |
| --- | --- | --- | --- | --- |
| Statistical methods | 12 | (*a*) Describe all statistical methods, including those used to control for confounding | 3-4 | In the first step, the odds ratio (OR) between the identification status of each UPSIT-TC odor and the clinical scores was calculated using logistic regression analysis. Odors showing a statistically significant odds ratio between their identification status and clinical scores were grouped as symptom-specific odors. The identification of a symptom-specific odor was scored as one, while misidentification of this smell was scored as zero. The symptom-specific odor score was calculated as the sum of the identification status of odors that showed a statistically significant odds ratio with the specific clinical score. For example, if the detection status of cedar, rubber tire, and peach odors showed a significant odds ratio (OR) with the severity of NMSS gastrointestinal symptoms, these three odors were classified as NMSS gastrointestinal symptom-specific odors. If a patient’s UPSIT-TC score was 13 and they correctly identified all three symptom-specific odors during the test, their NMSS gastrointestinal symptom-specific odor score would be three. This demonstrates that symptom-specific odors vary for different PD clinical symptoms. The second-step analysis was conducted only when the number of symptom-specific odors for the analyzed PD symptom exceeded one.  In the second step, correlation analysis was applied to calculate Pearson's r between the UPSIT-TC score and the analyzing clinical score, and the symptom-specific odor score and the analyzing clinical score.  In the final step, linear regression analysis was performed, with age, gender, disease duration, and LEDD as covariates, to explore the significance of the symptom-specific odor score and UPSIT-TC score in predicting the clinical scores that showed significance in the previous correlation analysis. |
|  |  | (*b*) Describe any methods used to examine subgroups and interactions |  | Not used. |
|  |  | (*c*) Explain how missing data were addressed |  | All eligible patients without missing data were consecutively included. |
|  |  | (*d*) *Cohort study*—If applicable, explain how loss to follow-up was addressed  *Case-control study*—If applicable, explain how matching of cases and controls was addressed  *Cross-sectional study*—If applicable, describe analytical methods taking account of sampling strategy |  | All eligible patients were consecutively included; therefore, no sampling strategy or weighting was applied. |
|  |  | (*e*) Describe any sensitivity analyses |  | No sensitivity analyses were performed. |
| Results | | | | |
| Participants | 13* | (a) Report numbers of individuals at each stage of study—eg numbers potentially eligible, examined for eligibility, confirmed eligible, included in the study, completing follow-up, and analysed | 4 | A total of 176 consecutive patients who met the inclusion criteria during the study period were included in the final analysis. |
|  |  | (b) Give reasons for non-participation at each stage |  | No non-participation occurred, as all eligible patients were consecutively included from the retrospective database. |
|  |  | (c) Consider use of a flow diagram |  | No flow diagram was provided |
| Descriptive data | 14* | (a) Give characteristics of study participants (eg demographic, clinical, social) and information on exposures and potential confounders | 4,9 | The average age of the participants was 67.1 ± 9.71 years, with 110 males and 66 females. The average disease duration was 4.69 ± 5.60 years. The mean score on the MDS-UPDRS scale was 51.57 ± 26.33, and the average LEDD was 518.73 ± 445.14 mg/day. Detailed participant data are presented in Table 1. |
|  |  | (b) Indicate number of participants with missing data for each variable of interest |  | There were no missing data for any variables of interest, as all eligible patients were included from a complete retrospective clinical database. |
|  |  | (c) *Cohort study*—Summarise follow-up time (eg, average and total amount) |  | Not applicable |
| Outcome data | 15* | *Cohort study*—Report numbers of outcome events or summary measures over time |  |  |
|  |  | *Case-control study—*Report numbers in each exposure category, or summary measures of exposure |  |  |
|  |  | *Cross-sectional study—*Report numbers of outcome events or summary measures | 4-6, 10-12 | ** Odors Associated with Motor and Non-Motor Severity…  ** Comparisons of Correlations Between UPSIT Total Score and Symptom-Specific Odor Scores with PD Clinical Symptoms  **Table 2-4 |
| Main results | 16 | (*a*) Give unadjusted estimates and, if applicable, confounder-adjusted estimates and their precision (eg, 95% confidence interval). Make clear which confounders were adjusted for and why they were included | 4-6, 10-12 | when age, gender, disease duration, and LEDD were included as covariates  ** Odors Associated with Motor and Non-Motor Severity…  ** Comparisons of Correlations Between UPSIT Total Score and Symptom-Specific Odor Scores with PD Clinical Symptoms  **Table 2-4 |
|  |  | (*b*) Report category boundaries when continuous variables were categorized |  | Not applicable |
|  |  | (*c*) If relevant, consider translating estimates of relative risk into absolute risk for a meaningful time period |  | Not applicable |

Continued on next page

| Other analyses | 17 | Report other analyses done—eg analyses of subgroups and interactions, and sensitivity analyses | 5-6 | Linear regression analysis was then performed using the PD symptom-specific odor score and UPSIT total score to predict the severity of motor and non-motor symptoms in the PD patients (Tables 4). Two separate models were established: Model 1 & 2 considered age, gender, disease duration, LEDD, and different odor scores as covariates.  When age, gender, disease duration, and LEDD were included as cofactors, the predictive power for MDS-UPDRS part III score, rigidity subscore, and MoCA score was observed in both models containing the UPSIT total score (MDS-UPDRS part III score: B = -0.342, p = 0.034; rigidity subscore: B = -0.152, p = 0.001; and MoCA score: B = 0.111, p = 0.032) and symptom-specific odor score (MDS-UPDRS part III score: B = -3.252, p = 0.004; rigidity subscore: B = -0.785, p < 0.001; and MoCA score: B = 0.958, p < 0.001). The linear regression analysis demonstrated significant predictive values for the MDS-UPDRS total score (B = -3.890, p < 0.001), MDS-UPDRS part II score (B = -1.836, p < 0.001), and NMSS gastrointestinal tract subscore (B = -1.347, p = 0.005) in the symptom-specific odor score model but not in the UPSIT total score model. |
| --- | --- | --- | --- | --- |
| Discussion | | | | |
| Key results | 18 | Summarise key results with reference to study objectives | 6 | This study analyzed the correlations between individual UPSIT odors and clinical symptoms of PD to explore the potential of using a brief olfactory test to assess PD severity. Seventeen odors—orange, sandalwood, rubber tire, peach, pizza, leather, smoke, bubble gum, watermelon, jasmine, turpentine, cedar, mint, fish, lemon, coffee, and pine—showed significant correlations with the severity of PD symptoms after adjusting for age and gender as covariates. When odors associated with specific PD symptoms were grouped into “symptom-specific odors,” the correlations were stronger and approached those observed with total UPSIT scores. This significance persisted even after considering additional covariates, including age, gender, disease duration, and LEDD in the linear regression models. However, the predictive associations of the total UPSIT score with the MDS-UPDRS total score, MDS-UPDRS Part II score, and the NMSS gastrointestinal domain score were no longer significant after these additional adjustments. |
| Limitations | 19 | Discuss limitations of the study, taking into account sources of potential bias or imprecision. Discuss both direction and magnitude of any potential bias | 7 | This study has several limitations. First, selection bias may be present due to the single-center design and the inclusion of only patients with a confirmed diagnosis of PD without a control group for comparison. This may limit the ability to determine whether selective odor loss is specific to PD patients. However, this study demonstrated the potential of using a simple olfactory identification test as a tool correlated with clinical symptoms after confirming a PD diagnosis. Second, all the enrolled patients were of Chinese ethnicity, and cultural differences may influence odor identification across different ethnicities [35, 36]. In addition, as this was a single-center study conducted at a tertiary referral center, the findings may not be fully generalizable to broader populations or different clinical settings. Further multi-ethnic studies are warranted. Third, the average MoCA score (24.13 ± 5.24) in this study was relatively low. Although we could not exclude the possibility of including some patients with cognitive decline, the normative MoCA score for the Taiwanese population in the same age group was 23.80 ± 4.57 in a previous study [37], and cognitive function was included as an important investigational outcome in this study. Fourth, as a retrospective observational clinical study, it was challenging to clarify the underlying pathophysiology. Future studies incorporating functional MRI, electrophysiological testing, post-mortem autopsies, or blood tests for genetic polymorphisms may provide greater insights into the relationship between selective odor identification deficits and PD clinical severity. |
| Interpretation | 20 | Give a cautious overall interpretation of results considering objectives, limitations, multiplicity of analyses, results from similar studies, and other relevant evidence | 8 | This is the first study to demonstrate the potential of developing a shortened olfactory identification test with better predictive ability for PD clinical severity than the complete UPSIT. Future studies are warranted to explore odors specific to PD clinical symptoms beyond the 17 significant odors identified in this study to create a new, more time-efficient tool. Such a tool could serve as a valuable method to predict the severity of both motor and non-motor symptoms in patients with PD in the future. |
| Generalisability | 21 | Discuss the generalisability (external validity) of the study results | 7 | This study has several limitations. First, selection bias may be present due to the single-center design and the inclusion of only patients with a confirmed diagnosis of PD without a control group for comparison. This may limit the ability to determine whether selective odor loss is specific to PD patients. However, this study demonstrated the potential of using a simple olfactory identification test as a tool correlated with clinical symptoms after confirming a PD diagnosis. Second, all the enrolled patients were of Chinese ethnicity, and cultural differences may influence odor identification across different ethnicities [35, 36]. In addition, as this was a single-center study conducted at a tertiary referral center, the findings may not be fully generalizable to broader populations or different clinical settings. Further multi-ethnic studies are warranted. |
| Other information | |  | | |
| Funding | 22 | Give the source of funding and the role of the funders for the present study and, if applicable, for the original study on which the present article is based | 8 | This study was funded by a grant from Taichung Veterans General Hospital (TCVGH-1144501B). |

*Give information separately for cases and controls in case-control studies and, if applicable, for exposed and unexposed groups in cohort and cross-sectional studies.

**Note:** An Explanation and Elaboration article discusses each checklist item and gives methodological background and published examples of transparent reporting. The STROBE checklist is best used in conjunction with this article (freely available on the Web sites of PLoS Medicine at http://www.plosmedicine.org/, Annals of Internal Medicine at http://www.annals.org/, and Epidemiology at http://www.epidem.com/). Information on the STROBE Initiative is available at www.strobe-statement.org.
