# Supplementary material for: Odor‐Specific Symptoms in Parkinson’s Disease—UPSIT‐TC Odor Identification Status Versus Clinical Severity
Source: Parkinsons Dis. 2026 Oct 22;2026:9572752. doi: 10.1155/padi/9572752 (PMC13687933; doi:10.1155/padi/9572752)
Supplement: Supplementary file 2 [file PADI-2026-9572752-s002.docx]

**Supplementary Table 1.** Full list of nominal associations between clinical symptom severity and individual UPSIT-TC odor identification

| Clinical domain | Symptom severity score | Associated odor item | OR for correct odor identification | p value | q value | Age/sex-adjusted p value |
| --- | --- | --- | --- | --- | --- | --- |
| Overall motor/non-motor severity | MDS-UPDRS Total | Pizza | 0.981 | 0.032 | 0.162 | 0.024 |
| Overall motor/non-motor severity | MDS-UPDRS Total | Bubble gum | 0.986 | 0.037 | 0.162 | 0.079 |
| Overall motor/non-motor severity | MDS-UPDRS Total | Mint | 0.986 | 0.020 | 0.134 | 0.042 |
| Overall motor/non-motor severity | MDS-UPDRS Total | Sandalwood | 0.982 | 0.005 | 0.070 | 0.040 |
| Overall motor/non-motor severity | MDS-UPDRS Total | Leather | 0.983 | 0.008 | 0.070 | 0.037 |
| Overall motor/non-motor severity | MDS-UPDRS Total | Cedar | 0.986 | 0.041 | 0.162 | 0.077 |
| Overall motor/non-motor severity | MDS-UPDRS Total | Rubber tire | 0.976 | <0.001 | 0.015 | 0.004 |
| Overall motor/non-motor severity | MDS-UPDRS Total | Peach | 0.981 | 0.007 | 0.070 | 0.024 |
| Overall motor/non-motor severity | MDS-UPDRS Total | Orange | 0.985 | 0.036 | 0.162 | 0.050 |
| Overall motor/non-motor severity | MDS-UPDRS Total | Smoke | 0.982 | 0.009 | 0.070 | 0.032 |
| Non-motor experiences of daily living | MDS-UPDRS Part I | Sandalwood | 0.951 | 0.042 | 0.418 | 0.128 |
| Non-motor experiences of daily living | MDS-UPDRS Part I | Leather | 0.945 | 0.024 | 0.316 | 0.067 |
| Non-motor experiences of daily living | MDS-UPDRS Part I | Rubber tire | 0.926 | 0.003 | 0.110 | 0.007 |
| Non-motor experiences of daily living | MDS-UPDRS Part I | Smoke | 0.943 | 0.023 | 0.316 | 0.050 |
| Motor experiences of daily living | MDS-UPDRS Part II | Bubble gum | 0.948 | 0.010 | 0.081 | 0.024 |
| Motor experiences of daily living | MDS-UPDRS Part II | Mint | 0.965 | 0.043 | 0.189 | 0.088 |
| Motor experiences of daily living | MDS-UPDRS Part II | Sandalwood | 0.944 | 0.001 | 0.028 | 0.015 |
| Motor experiences of daily living | MDS-UPDRS Part II | Leather | 0.956 | 0.016 | 0.095 | 0.072 |
| Motor experiences of daily living | MDS-UPDRS Part II | Rubber tire | 0.933 | <0.001 | 0.023 | 0.006 |
| Motor experiences of daily living | MDS-UPDRS Part II | Lilac | 0.959 | 0.017 | 0.095 | 0.014 |
| Motor experiences of daily living | MDS-UPDRS Part II | Peach | 0.944 | 0.006 | 0.068 | 0.023 |
| Motor experiences of daily living | MDS-UPDRS Part II | Orange | 0.939 | 0.007 | 0.068 | 0.009 |
| Motor experiences of daily living | MDS-UPDRS Part II | Smoke | 0.960 | 0.033 | 0.167 | 0.115 |
| Motor examination | MDS-UPDRS Part III | Pizza | 0.966 | 0.028 | 0.149 | 0.028 |
| Motor examination | MDS-UPDRS Part III | Bubble gum | 0.974 | 0.031 | 0.149 | 0.078 |
| Motor examination | MDS-UPDRS Part III | Mint | 0.974 | 0.024 | 0.149 | 0.043 |
| Motor examination | MDS-UPDRS Part III | Sandalwood | 0.977 | 0.041 | 0.149 | 0.236 |
| Motor examination | MDS-UPDRS Part III | Leather | 0.977 | 0.041 | 0.149 | 0.140 |
| Motor examination | MDS-UPDRS Part III | Rubber tire | 0.968 | 0.006 | 0.149 | 0.057 |
| Motor examination | MDS-UPDRS Part III | Peach | 0.972 | 0.020 | 0.149 | 0.055 |
| Motor examination | MDS-UPDRS Part III | Jasmine | 0.974 | 0.036 | 0.149 | 0.058 |
| Motor examination | MDS-UPDRS Part III | Watermelon | 0.968 | 0.011 | 0.149 | 0.025 |
| Motor examination | MDS-UPDRS Part III | Baby powder | 0.976 | 0.034 | 0.149 | 0.093 |
| Motor examination | MDS-UPDRS Part III | Smoke | 0.973 | 0.022 | 0.149 | 0.074 |
| Motor examination | Bradykinesia | Bubble gum | 0.948 | 0.012 | 0.365 | 0.022 |
| Motor examination | Bradykinesia | Mint | 0.961 | 0.044 | 0.365 | 0.070 |
| Motor examination | Axial | Menthol | 0.919 | 0.041 | 0.321 | 0.049 |
| Motor examination | Axial | Mint | 0.912 | 0.033 | 0.321 | 0.070 |
| Motor examination | Axial | Sandalwood | 0.914 | 0.028 | 0.321 | 0.209 |
| Motor examination | Axial | Licorice | 0.917 | 0.048 | 0.321 | 0.056 |
| Motor examination | Axial | Rubber tire | 0.902 | 0.017 | 0.321 | 0.127 |
| Motor examination | Axial | Orange | 0.880 | 0.017 | 0.321 | 0.019 |
| Motor examination | Rigidity | Pizza | 0.884 | 0.015 | 0.086 | 0.019 |
| Motor examination | Rigidity | Menthol | 0.922 | 0.041 | 0.119 | 0.086 |
| Motor examination | Rigidity | Motor oil | 0.916 | 0.048 | 0.119 | 0.085 |
| Motor examination | Rigidity | Banana | 0.922 | 0.044 | 0.119 | 0.060 |
| Motor examination | Rigidity | Sandalwood | 0.917 | 0.039 | 0.119 | 0.150 |
| Motor examination | Rigidity | Leather | 0.916 | 0.027 | 0.119 | 0.061 |
| Motor examination | Rigidity | Onion | 0.923 | 0.047 | 0.119 | 0.131 |
| Motor examination | Rigidity | Rubber tire | 0.874 | 0.001 | 0.022 | 0.009 |
| Motor examination | Rigidity | Turpentine | 0.894 | 0.006 | 0.057 | 0.014 |
| Motor examination | Rigidity | Peach | 0.900 | 0.011 | 0.070 | 0.017 |
| Motor examination | Rigidity | Jasmine | 0.878 | 0.003 | 0.037 | 0.005 |
| Motor examination | Rigidity | Watermelon | 0.866 | <0.001 | 0.022 | 0.002 |
| Motor examination | Rigidity | Paint thinner | 0.921 | 0.042 | 0.119 | 0.054 |
| Motor examination | Rigidity | Smoke | 0.921 | 0.040 | 0.119 | 0.092 |
| Motor examination | Rigidity | Natural gas | 0.919 | 0.034 | 0.119 | 0.073 |
| Motor examination | Rigidity | Peanut | 0.896 | 0.009 | 0.070 | 0.009 |
| Disease stage | H&Y stage | Sandalwood | 0.585 | 0.013 | 0.265 | 0.163 |
| Disease stage | H&Y stage | Leather | 0.621 | 0.029 | 0.285 | 0.134 |
| Disease stage | H&Y stage | Cedar | 0.572 | 0.022 | 0.285 | 0.049 |
| Disease stage | H&Y stage | Rubber tire | 0.560 | 0.009 | 0.265 | 0.119 |
| Medication burden | LEDD | Coconut | 0.999 | 0.023 | 0.368 | 0.018 |
| Medication burden | LEDD | Lilac | 0.999 | 0.014 | 0.368 | 0.010 |
| Medication burden | LEDD | Peach | 0.999 | 0.028 | 0.368 | 0.016 |
| Non-motor symptom burden | NMSS Total | Rubber tire | 0.986 | 0.008 | 0.321 | 0.024 |
| Non-motor symptom burden | NMSS Total | Peach | 0.987 | 0.030 | 0.455 | 0.074 |
| Non-motor symptom burden | NMSS Total | Smoke | 0.989 | 0.042 | 0.455 | 0.092 |
| Non-motor symptom burden | NMSS Total | Soap | 0.990 | 0.046 | 0.455 | 0.036 |
| Non-motor symptom domain | NMSS Gastrointestinal | Mint | 0.938 | 0.019 | 0.193 | 0.025 |
| Non-motor symptom domain | NMSS Gastrointestinal | Leather | 0.947 | 0.043 | 0.290 | 0.077 |
| Non-motor symptom domain | NMSS Gastrointestinal | Coconut | 0.923 | 0.044 | 0.290 | 0.044 |
| Non-motor symptom domain | NMSS Gastrointestinal | Cedar | 0.926 | 0.019 | 0.193 | 0.028 |
| Non-motor symptom domain | NMSS Gastrointestinal | Rubber tire | 0.918 | 0.003 | 0.106 | 0.008 |
| Non-motor symptom domain | NMSS Gastrointestinal | Peach | 0.928 | 0.015 | 0.193 | 0.024 |
| Non-motor symptom domain | NMSS Sexual | Sandalwood | 0.906 | 0.050 | 0.435 | 0.172 |
| Non-motor symptom domain | NMSS Sexual | Leather | 0.891 | 0.048 | 0.435 | 0.094 |
| Non-motor symptom domain | NMSS Sexual | Rubber tire | 0.836 | 0.006 | 0.254 | 0.034 |
| Non-motor symptom domain | NMSS Sexual | Lemon | 1.135 | 0.015 | 0.297 | 0.012 |
| Non-motor symptom domain | NMSS Sleep/fatigue | Rubber tire | 0.949 | 0.010 | 0.411 | 0.029 |
| Non-motor symptom domain | NMSS Urinary | Fish | 0.812 | 0.020 | 0.371 | 0.027 |
| Non-motor symptom domain | NMSS Urinary | Chocolate | 0.950 | 0.041 | 0.371 | 0.065 |
| Non-motor symptom domain | NMSS Urinary | Rubber tire | 0.945 | 0.028 | 0.371 | 0.105 |
| Non-motor symptom domain | NMSS Urinary | Peach | 0.924 | 0.010 | 0.371 | 0.025 |
| Non-motor symptom domain | NMSS Perceptual | Coffee | 1.201 | 0.020 | 0.797 | 0.019 |
| Cognition | MoCA | Bubble gum | 1.097 | 0.007 | 0.059 | 0.012 |
| Cognition | MoCA | Menthol | 1.075 | 0.017 | 0.096 | 0.006 |
| Cognition | MoCA | Mint | 1.104 | 0.003 | 0.050 | 0.007 |
| Cognition | MoCA | Sandalwood | 1.088 | 0.006 | 0.059 | 0.217 |
| Cognition | MoCA | Rubber tire | 1.128 | <0.001 | 0.013 | 0.010 |
| Cognition | MoCA | Peach | 1.101 | 0.006 | 0.059 | 0.049 |
| Cognition | MoCA | Pineapple | 1.077 | 0.032 | 0.162 | 0.320 |
| Cognition | MoCA | Smoke | 1.082 | 0.017 | 0.096 | 0.111 |
| Neuropsychiatric symptoms | NPI Total | Licorice | 0.952 | 0.033 | 0.653 | 0.039 |
| Neuropsychiatric symptoms | NPI Total | Rubber tire | 0.941 | 0.007 | 0.281 | 0.018 |
| Impulse-control symptoms | QUIP-RS | Pine | 1.189 | 0.012 | 0.490 | 0.013 |
| Sleep quality | PDSS | Leather | 1.022 | 0.003 | 0.115 | 0.009 |
| Sleep quality | PDSS | Rubber tire | 1.017 | 0.014 | 0.283 | 0.033 |
| Quality of life | PDQ-39 SI | Sandalwood | 0.980 | 0.021 | 0.424 | 0.050 |
| Quality of life | PDQ-39 SI | Cedar | 0.980 | 0.043 | 0.550 | 0.051 |
| Quality of life | PDQ-39 SI | Rubber tire | 0.977 | 0.010 | 0.394 | 0.014 |

Values are odds ratios estimated from logistic regression models examining the association between each clinical severity score and correct identification of each UPSIT-TC odor item. Displayed p values are rounded to three decimal places. The q values were calculated using the Benjamini–Hochberg false discovery rate procedure across all 40 odor-specific models for each clinical measure. Age/sex-adjusted p values were obtained from models additionally adjusted for age and sex. Results in this supplementary table represent the full list of nominally significant odor–symptom associations and should be interpreted as exploratory. UPSIT-TC, Traditional Chinese version of the University of Pennsylvania Smell Identification Test; OR, Odds ratio; LEDD, levodopa equivalent daily dose; MDS-UPDRS, Movement Disorder Society Unified Parkinson’s Disease Rating Scale; H&Y stage, Hoehn and Yahr stage; NMSS, Non-Motor Symptoms Scale; MoCA, Montreal Cognitive Assessment; NPI, Neuropsychiatric Inventory; QUIP-RS, Questionnaire for Impulsive-Compulsive Disorders in Parkinson’s Disease–Rating Scale; PDSS, Parkinson’s Disease Sleep Scale; PDQ-39 SI, 39-item Parkinson’s Disease Questionnaire summary index; Bradykinesia subscore, MDS-UPDRS Part III items 3.2, 3.4–3.9, and 3.14; Rigidity subscore, MDS-UPDRS Part III item 3.3; Axial subscore, MDS-UPDRS Part III items 3.1 and 3.9–3.13

**Supplementary Table 2.** Correlations between clinical severity scores and olfactory scores (N = 176)

| Clinical measure | UPSIT-TC total score | | Symptom-specific odor score | |
| --- | --- | --- | --- | --- |
|  | **r/ρ** | ***p* value** | **r/ρ** | ***p* value** |
| MDS-UPDRS |  |  |  |  |
| Total | -0.262 | <0.001* | -0.403 | <0.001*^ |
| Part I | -0.143 | 0.058 | -0.264 | <0.001*^ |
| Part II | -0.245 | 0.001* | -0.400 | <0.001*^ |
| Part III | -0.248 | <0.001*^ | -0.342 | <0.001*^ |
| Bradykinesia | -0.159 | 0.035 | -0.227 | 0.002*^ |
| Rigidity | -0.311 | <0.001*^ | -0.401 | <0.001*^ |
| Axial | -0.169 | 0.025 | -0.307 | <0.001*^ |
| H&Y stage | -0.173 | 0.022 | -0.283 | <0.001*^ |
| LEDD | -0.154 | 0.041*^ | -0.263 | <0.001*^ |
| MoCA | 0.257 | <0.001*^ | 0.410 | <0.001*^ |
| NMSS |  |  |  |  |
| Total | -0.135 | 0.074 | -0.266 | <0.001*^ |
| Gastrointestinal tract | -0.233 | 0.002*^ | -0.326 | <0.001*^ |
| Urinary | -0.094 | 0.216 | -0.281 | <0.001*^ |
| Sexual function | -0.090 | 0.235 | -0.139 | 0.067 |
| NPI Total | -0.124 | 0.100 | -0.252 | <0.001*^ |
| PDSS | 0.126 | 0.095 | 0.257 | <0.001*^ |
| PDQ-39 SI | -0.126 | 0.096 | -0.252 | <0.001*^ |

Correlation analyses were performed using Pearson’s correlation coefficient (r), except for H&Y stage, for which Spearman’s rank correlation coefficient (ρ) was used. *Association remained significant after adjustment for age and sex (p<0.05); ^Association remained significant after adjustment for age, sex, disease duration, and LEDD (p<0.05), except when LEDD was the clinical measure, in which case adjustment included age, sex, and disease duration only. PD, Parkinson’s disease; UPSIT-TC, Traditional Chinese version of the University of Pennsylvania Smell Identification Test; N, number of patients; MDS-UPDRS, Movement Disorder Society Unified Parkinson’s Disease Rating Scale; Bradykinesia subscore, MDS-UPDRS Part III items 3.2, 3.4–3.9, and 3.14; Rigidity subscore, MDS-UPDRS Part III item 3.3; Axial subscore, MDS-UPDRS Part III items 3.1 and 3.9–3.13; H&Y stage, Hoehn and Yahr stage; MoCA, Montreal Cognitive Assessment; NMSS, Non-Motor Symptoms Scale; NPI, Neuropsychiatric Inventory; PDSS, Parkinson’s Disease Sleep Scale; PDQ-39 SI, 39-item Parkinson’s Disease Questionnaire summary index.
